# Change in kidney volume growth rate and renal outcomes of tolvaptan treatment in autosomal dominant polycystic kidney disease: post-hoc analysis of TEMPO 3:4 trial

**DOI:** 10.1007/s10157-024-02589-1

**Published:** 2025-01-02

**Authors:** Eiji Higashihara, Miyuki Matsukawa, Huan Jiang

**Affiliations:** 1 Department of Urology, Kyorin University School of Medicine, 6-20-2 Shinkawa, Mitaka, Tokyo 181-8611 Japan; 2 Medical Affairs, Otsuka Pharmaceutical Co., Ltd., Osaka, Japan; 3 Otsuka Pharmaceutical Development and Commercialization, Princeton, NJ USA

**Keywords:** Autosomal dominant polycystic kidney disease, Estimated glomerular filtration rate, Estimated height-adjusted total kidney volume growth rate, Tolvaptan, Total kidney volume

## Abstract

**Background:**

Despite of long-lasting tolvaptan treatment, individual renal outcomes are unclear in autosomal dominant polycystic kidney disease (ADPKD). This post-hoc analysis of the TEMPO 3:4 trial aimed to evaluate the predictability of estimated height-adjusted total kidney volume growth rate (eHTKV-α) on renal outcomes.

**Methods:**

In TEMPO 3:4, 1445 patients with ADPKD were randomised to tolvaptan or placebo for 3 years. The present analysis included patients with total kidney volume (TKV) data available at baseline and month 12 (tolvaptan, *n* = 812; placebo, *n* = 453); tolvaptan-assigned patients were grouped into quartiles based on percent change in eHTKV-α from baseline at 1 year. Clinical parameters were compared between quartiles, and regression analyses evaluated the predictive value of 1-year percent change in eHTKV-α and other factors on annual changes in TKV and estimated GFR (eGFR) over 3 years.

**Results:**

Trend tests identified significant differences between quartiles for several baseline parameters. Multivariate regression models confirmed that 1-year percent change in eHTKV-α was a significant predictor of annual changes in both TKV and eGFR over 3 years. Other significant predictors of annual changes in TKV and eGFR over 3 years were sex, age and body mass index, and first-year change in eGFR, race and baseline eGFR, respectively. Predicting factors using urine osmolality and plasma copeptin levels were not significant by backward stepwise selection analysis.

**Conclusions:**

1-year percent change in eHTKV-α is useful biomarker to identify treatment good responders and may be utilized for early estimate of trial outcomes of new drugs in ADPKD.

**Supplementary Information:**

The online version contains supplementary material available at 10.1007/s10157-024-02589-1.

## Introduction

Autosomal dominant polycystic kidney disease (ADPKD) is an inherited renal disorder that is caused by *PKD1* or *PKD2* mutations, affects approximately 1 in 1000 live births, and is characterised by the relentless development of renal cysts. Continuous renal cyst expansion anatomically disrupts and obstructs renal tubules, leading to ultimately to end-stage renal disease (ESRD) in a majority of patients [1, 2].

Adenosine-3′,5′-cyclic monophosphate (cAMP) plays a major role in chloride secretion into the cysts and promotes increased proliferation of the cyst-lining cells [3]. Vasopressin V_2_ receptor is a major cAMP stimulator in renal tubules [4], and vasopressin V_2_ receptor antagonists lowered renal cAMP, inhibited renal cystogenesis and disease progression in animal models of the human polycystic kidney disease [5].

Based on this evidence, two global phase 3 trials were performed, TEMPO 3:4 [6] and REPRISE [7]. Tolvaptan reduced the rates of total kidney volume (TKV) growth and estimated glomerular filtration rate (eGFR) decline in patients with early ADPKD in the TEMPO 3:4 study, and reduced the rate of eGFR decline in patients with later-stage ADPKD in the REPRISE study. Based on the results of both studies, tolvaptan was approved for the treatment of ADPKD in many countries, with the eligibility criterion of “rapidly progressing disease” [8–10]. Given the long-term nature of tolvaptan treatment, its association with polyuria and other possible adverse effects, it is also important to select good treatment responders and ensure that patients taking tolvaptan are likely to benefit from treatment. Monitoring individual treatment effects by routine measurement of eGFR slope is recommended [11]; however, the lack of sufficient pre-treatment data in most patients and the frequent natural occurrence of non-linear eGFR slopes may obscure the comparison [12].

To ensure individual treatment efficacy, several studies have sought to identify factors that may predict tolvaptan efficacy. Post hoc analyses of TEMPO 3:4 data have found that greater responses to tolvaptan were achieved in patients with higher urine osmolality at baseline, greater suppression of urine osmolality from baseline to week 3 of treatment, those with better eGFR at baseline [13], higher plasma copeptin levels at baseline and greater increases in plasma copeptin from baseline to week 3 [14].

Previous research has shown that renal enlargement in ADPKD exhibits exponential growth [15]. Height-adjusted TKV (HtTKV) growth rate (eHTKV-α) estimated by using 1 TKV observation and age is more stable than the TKV growth rate estimated by using 2 TKV observations because the HtTKV measurement error is spread to 20 or more years in calculation of eHTKV-α but the error is spread to only few years in the latter case [16, 17]. More recently, the equation used to calculate eHTKV-α was refined using large case number [18]. Mayo-imaging classification identified groups with a high risk of ESRD [16]. Likewise, the area under the receiver-operating characteristic curves using eHTKV-α to predict ESRD was high (0.89 ± 0.04) [19]. In addition to the identification of patients at high risk of renal progression, treatment-induced long-term renal outcomes may be predicted by changes in eHTKV-α. Indeed, a small, single-centre, non-comparative study showed that a larger decrease in eHTKV-α during the first year of tolvaptan therapy was associated with a slower decline in eGFR over 5 years of treatment, indicating that 1-year change in eHTKV-α could be used to identify poor and good treatment responders [20]. To further explore this hypothesis in a larger ADPKD dataset, the present study aimed to define good treatment response by characterising the relationship between 1-year change in eHTKV-α and 3-year eGFR and TKV outcomes in TEMPO 3:4, and to compare the predictive value of eHTKV-α with other factors associated with tolvaptan efficacy in ADPKD.

## Materials and methods

### TEMPO 3:4 study design

This was a post hoc analysis of the TEMPO 3:4 study (ClinicalTrials.gov identifier, NCT00428948) [6]. TEMPO 3:4 was a phase 3, multicentre, double-blind, placebo-controlled study that assessed the efficacy and safety of tolvaptan in patients with ADPKD. Eligible patients were aged 18–50 years, with diagnosed ADPKD, TKV ≥ 750 ml and estimated creatine clearance ≥ 60 ml/min; and 1445 patients with ADPKD were enrolled worldwide between January 2007 and January 2009. After an initial 3-week dose-escalation phase, patients continued tolvaptan treatment at the highest tolerated dose through year 3 (Supplementary CONSORT check list).

The TEMPO 3:4 study was conducted in accordance with the principles of the Declaration of Helsinki and the International Conference of Harmonisation Good Clinical Practice Guidelines, with an institutional review board or ethics committee at each site approving the study protocol before initiation. All patients provided written informed consent to participate.

### Study parameters

Evaluations relevant to the present analysis were TKV and eGFR (calculated using the Chronic Kidney Disease Epidemiology Collaboration equation). In the TEMPO 3:4 primary analysis, eGFR assessments were conducted at baseline, randomisation, weekly during the dose-escalation phase, every 4 months during the 3-year treatment period, and twice between weeks 1–6 after treatment ended. TKV assessments were conducted at baseline and at 1, 2 and 3 years (± every 2 weeks) during the treatment period [6].

In the present analysis, eHTKV-α was calculated at baseline and year 1, and the percent change in eHTKV-α was measured between these timepoints. eHTKV-α (%/year) was calculated using the equation below, where HtTKV_t_ denotes HtTKV at age t (in years) and was expressed as HtTKV_t_ = 130 × (1 + eHTKV-α/100)^t^ [18].

$$\text{eHTKV-}\alpha =\left({10}^{\left(\frac{Log10\left(\frac{{HtTKV}_{t}}{130}\right)}{t}\right)}-1\right)\times 100$$

### Post hoc analyses

This post hoc analysis included all patients in TEMPO 3:4 who had TKV data available to calculate eHTKV-α at baseline and year 1. Tolvaptan-treated patients were further grouped into quartiles (TQ1–TQ4) based on the percent change in eHTKV-α from baseline at 1 year.

The primary objective of this study was to examine the relationships between the change in eHTKV-α from baseline at 1 year and annual changes in TKV and eGFR during the 3-year treatment period. The annual change in TKV was calculated as the slope of a regression model over time on TKV values obtained at baseline, and years 1, 2 and 3. The annual change in eGFR was calculated as the slope of a regression model over time across all on-treatment eGFR values from week 3 through year 3.

Secondary analyses aimed to determine whether patient demographics and/or clinical parameters differed significantly between tolvaptan eHTKV-α quartiles, and between tolvaptan- and placebo-assigned patients in TEMPO 3:4. Further analyses evaluated the predictive value of 1-year eHTKV-α change and these other variables on annual changes in TKV and eGFR over 3 years.

### Statistical analysis

Data are summarised using descriptive statistics, including the means, standard deviation (SD), standard error (SE), medians and quartiles for continuous variables, and number of patients and percentages for categorical variables. Log-transformed TKV data were used to calculate annual changes in TKV, which were presented after conversion to natural numbers. To compare patient demographics and clinical parameters between tolvaptan eHTKV-α quartiles (TQ1–TQ4), a Cochran-Armitage trend test was used for categorical variables, and an analysis of variance (ANOVA) trend test was used for continuous variables. Corresponding comparisons between the overall tolvaptan and placebo groups were based on Chi-squared tests for categorical variables and two-sample t-tests for continuous variables.

Correlations were calculated to determine the associations between change in eHTKV-α from baseline at 1 year and annual changes in TKV and eGFR over 3 years (expressed as Pearson correlation coefficients [ρ] and 95% confidence intervals [CI]). The predictive value of percent change in eHTKV-α from baseline at 1 year on annual changes in TKV and eGFR was tested in each of the tolvaptan eHTKV-α quartiles, and was compared between tolvaptan and placebo groups. Statistical comparisons of annual TKV and eGFR change between tolvaptan eHTKV-α quartiles, and between tolvaptan and placebo groups, were based on ANOVA trend tests.

Univariate and multivariate regression analyses were conducted to determine the predictive value of other factors on the annual changes in TKV and eGFR over 3 years (expressed as standardised regression coefficients [St. ß] and *P* values). In order to select covariates that provide the simplest and most effective model for prediction, backward stepwise selection was applied to the multivariate model as a variable reduction method. All statistical analyses were performed using SAS version 9.4, and *P* < 0.05 was considered statistically significant.

## Results

### Patient characteristics by tolvaptan eHTKV-α quartile

In TEMPO 3:4, 1445 patients with ADPKD were randomised to receive tolvaptan (*n* = 961) or matching placebo (*n* = 484). Of these, 812 tolvaptan-assigned patients and 453 placebo-assigned patients had TKV data available to calculate eHTKV-α at baseline and year 1, and were subsequently included in this analysis. The 812 tolvaptan-treated patients were further divided into quartiles (TQ1–TQ4; each *n* = 203) based on percent change in eHTKV-α from baseline at 1 year.

Baseline patient demographic and clinical characteristics of the TEMPO 3:4 trial population have been reported previously [6]. For the present study, key patient demographics and clinical parameters by tolvaptan eHTKV-α quartile are summarised in Table 1 and Supplementary Table S1.

**Table 1:** Patient baseline demographics and clinical parameters by tolvaptan eHTKV-α quartile

	Tolvaptan^a^						Placebo^a^	
	All (*n* = 812)	Tolvaptan eHTKV-α quartile				*P* value^b^	All (*n* = 453)	*P* value^c^
		TQ1 (*n* = 203)	TQ2 (*n* = 203)	TQ3 (*n* = 203)	TQ4 (*n* = 203)			
Male sex, *n* (%)	423 (52.1)	72 (35.5)	98 (48.3)	112 (55.2)	141 (69.5)	** < 0.001**	234 (51.7)	0.9
Age (years), mean ± SD	38.8 ± 6.9	38.8 ± 6.9	39.1 ± 7.0	38.9 ± 6.9	38.3 ± 7.0	0.6	39.1 ± 7.1	0.5
TKV parameters								
TKV (ml), *n*	812	203	203	203	203		453	
Mean ± SD	1717.3 ± 912.4	1415.9 ± 579.1	1789.7 ± 820.7	1830.1 ± 992.5	1833.7 ± 1107.1	** < 0.001**	1677.0 ± 885.2	0.4
HtTKV (ml/m), *n*	812	203	203	203	203		453	
Mean ± SD	986.6 ± 511.6	827.2 ± 326.1	1026.7 ± 450.6	1048.2 ± 558.6	1044.5 ± 628.7	** < 0.001**	963.5 ± 490.0	0.4
eHTKV-α (%/year), *n*	812	203	203	203	203		453	
Mean ± SD	5.2 ± 1.5	4.8 ± 1.5	5.3 ± 1.4	5.3 ± 1.6	5.3 ± 1.5	**0.001**	5.1 ± 1.6	0.6
Change in eHTKV-α from baseline at 1 year (%), *n*	812	203	203	203	203		453	
Range		< − 6.3	− 6.3 to < − 3.7	− 3.7 to < − 1.2	≥ − 1.2			
Mean ± SD	− 3.7 ± 4.4	− 9.2 ± 2.6	− 4.9 ± 0.8	− 2.4 ± 0.7	1.7 ± 2.9	** < 0.001**	− 0.5 ± 4.6	** < 0.001**
Kidney function parameters								
Serum creatinine (mg/dl), *n*	809	203	202	201	203		451	
Mean ± SD	1.1 ± 0.3	1.0 ± 0.3	1.0 ± 0.3	1.1 ± 0.3	1.1 ± 0.3	** < 0.001**	1.0 ± 0.3	0.5
eGFR (ml/min per 1.73 m^2^), *n*	809	203	202	201	203		451	
Mean ± SD	80.7 ± 20.9	83.8 ± 19.3	81.2 ± 20.1	78.0 ± 20.9	79.6 ± 22.7	**0.04**	81.7 ± 22.8	0.4
Urine osmolality								
At baseline (mOsm/kg), *n*	797	197	199	199	202		448	
Mean ± SD	489.1 ± 174.7	522.5 ± 169.5	486.2 ± 166.1	469.6 ± 177.3	478.4 ± 182.0	**0.01**	507.1 ± 188.0	0.09
Change from baseline at week 3 (mOsm/Kg), n	778	194	191	195	198		441	
Mean ± SD	−296.6 ± 186.8	−326.5 ± 185.7	−284.7 ± 174.5	−275.1 ± 197.5	−299.9 ± 185.8	**0.04**	−63.4 ± 180.2	< **0.001**
Plasma copeptin								
At baseline (pmol/l), *n*	679	173	176	170	160		434	
Mean ± SD	8.5 ± 10.8	8.7 ± 13.4	9.3 ± 13.7	7.8 ± 6.4	8.3 ± 7.0	0.6	10.4 ± 22.0	0.09
Change from baseline at week 3 (pmol/l), *n*	590	156	147	149	138		391	
Mean ± SD	15.2 ± 16.2	16.8 ± 22.6	14.3 ± 16.5	16.0 ± 13.3	13.5 ± 7.5	0.3	− 1.9 ± 19.1	** < 0.001**
BP parameters								
Hypertension at age < 35 years, *n*	654	151	172	165	166		363	
* n* (%)	413 (63.1)	87 (57.6)	107 (62.2)	105 (63.6)	114 (68.7)	**0.04**	210 (57.9)	0.1

ANOVA, analysis of variance; BP, blood pressure; eGFR, estimated glomerular filtration rate; eHTKV-α, estimated height-adjusted total kidney volume growth rate; HtTKV, height-adjusted total kidney volume; SD, standard deviation; TKV, total kidney volume; TQ, tolvaptan eHTKV-α quartile

^a^Analyses included all patients in TEMPO 3:4 with TKV data available to calculate eHTKV-α at baseline and year 1; tolvaptan-assigned patients were further divided into quartiles (TQ1–TQ4) based on percent change in eHTKV-α from baseline at 1 year

^b^Statistical comparisons between tolvaptan eHTKV-α quartiles were based on Cochran Armitage tests for categorical variables and ANOVA trend tests for continuous variables

^c^Statistical comparisons between the overall tolvaptan and placebo treatment groups were based on Chi-squared tests for categorical variables and two-sample t-tests for continuous variables

Data are for parameters recorded at baseline, unless otherwise specified. Statistically significant values are indicated in bold

The proportion of male patients increased from TQ1 to TQ4 (*P* < 0.001; Table 1). Conversely, there were no significant between-quartile differences for baseline age. Baseline TKV and HtTKV increased from TQ1 to TQ4 (both *P* < 0.001).

Serum creatinine levels at baseline were significantly different between quartiles (*P* < 0.001); however, absolute differences were small across quartiles (Table 1). Baseline eGFR was highest in TQ1 and lowest in TQ3 (*P* = 0.04). Similarly, baseline urine osmolality was highest in TQ1 and lowest in TQ3 (*P* = 0.01) and mean change in urine osmolality from baseline at week 3 was highest in TQ1 and lowest in TQ3 (*P* = 0.04). Conversely, there were no significant between-quartile differences in mean plasma copeptin at baseline (*P* = 0.6) and mean change in plasma copeptin from baseline at week 3 (*P* = 0.3).

The proportion of patients with hypertension diagnosed before age 35 years at baseline was lowest in TQ1 and highest in TQ4 (*P* = 0.04; Table 1). As expected, comparisons between the overall tolvaptan and placebo groups showed that patient demographics and clinical parameters were similar between treatment arms, except for change in eHTKV-α from baseline at 1 year and change in urine osmolality and plasma copeptin from baseline at week 3 (all *P* < 0.001; Table 1 and Supplementary Table S1).

### Predictive value of eHTKV-α on renal outcomes

**Fig. 1:**
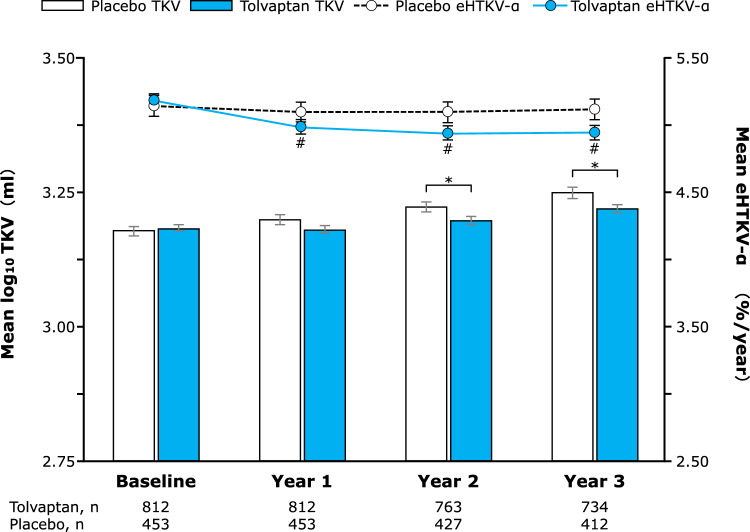
Mean TKV (bars) and eHTKV-α (points) over 3 years in the TEMPO 3:4 study. Analyses included all patients in TEMPO 3:4 with TKV data available to calculate eHTKV-α at baseline and years 1, 2 and 3; error bars represent standard errors. *P < 0.05 for comparisons of mean TKV between the tolvaptan and placebo groups at each time point; ^#^*P* < 0.001 for comparisons of mean eHTKV-α in the tolvaptan group versus baseline. eHTKV-α, estimated height-adjusted total kidney volume growth rate; TKV, total kidney volume

**Fig. 2:**
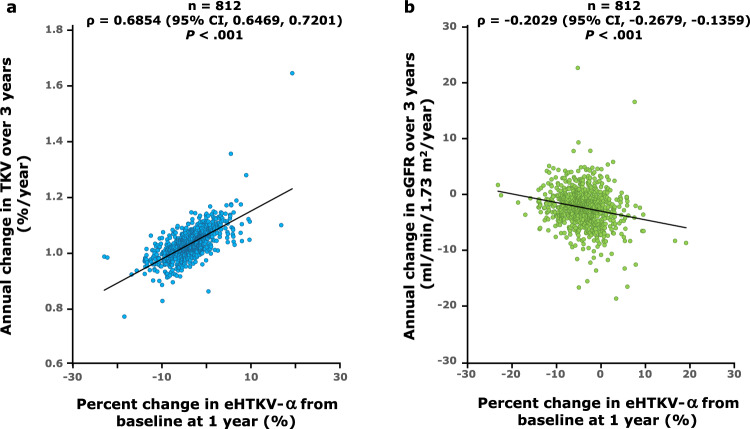
Correlation between percent change in eHTKV-α from baseline at 1 year and annual changes in **a** TKV and **b** eGFR over 3 years. Analyses included tolvaptan-assigned patients in TEMPO 3:4 with TKV data available to calculate eHTKV-α at baseline and year 1 (*n* = 812). CI, confidence interval; eGFR, estimated glomerular filtration rate; eHTKV-α, estimated height-adjusted total kidney volume growth rate; ρ, Pearson correlation coefficient; TKV, total kidney volume

**Fig. 3:**
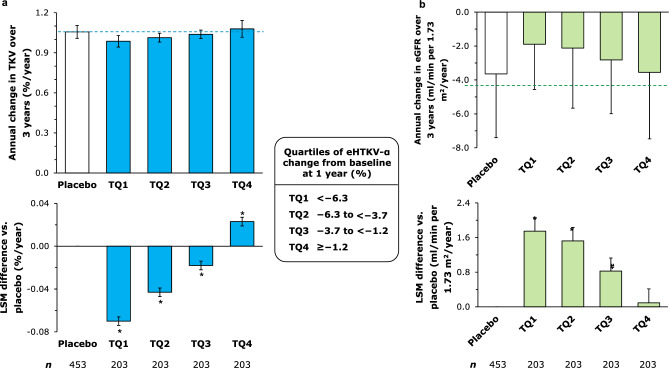
Relationship between annual changes in **a** TKV and **b** eGFR over 3 years and percent change in eHTKV-α from baseline at 1 year. Analyses included all patients in TEMPO 3:4 with TKV data available to calculate eHTKV-α at baseline and year 1; tolvaptan-assigned patients were further divided into quartiles (TQ1–TQ4) based on percent change in eHTKV-α from baseline at 1 year. Data presented in top panels are mean ± standard deviation; data presented in bottom panels are LSM ± standard error. *P < 0.001 for LSM difference in annual TKV or eGFR change over 3 years versus placebo; ^#^*P* = 0.007 for LSM difference in annual eGFR change over 3 years versus placebo. eGFR, estimated glomerular filtration rate; eHTKV-α, estimated height-adjusted total kidney volume growth rate; LSM, least squares mean; TKV, total kidney volume; TQ, tolvaptan eHTKV-α quartile

In TEMPO 3:4, tolvaptan therapy was associated with significant reductions in TKV growth versus placebo that coincided with significant reductions in eHTKV-α over 3 years (Fig. 1). Indeed, change in eHTKV-α from baseline at 1 year was strongly positively correlated with annual change in TKV over 3 years (*P* < 0.001; Fig. 2a). Compared with placebo, the annual change in TKV over 3 years was significantly smaller among tolvaptan-treated patients in TQ1–TQ3 and significantly larger among those in TQ4 (all *P* < 0.001; Table 2 and Fig. 3a).

**Table 2:** Relationship between annual changes in TKV and eGFR over 3 years and change in eHTKV-α from baseline at 1 year

		Tolvaptan^a^						Placebo^a^
		All (*n* = 812)	Tolvaptan eHTKV-α quartile				*P* value	All (n = 453)
			TQ1 (*n* = 203)	TQ2 (*n* = 203)	TQ3 (*n* = 203)	TQ4 (*n* = 203)		
Annual TKV change over 3 years								
Annual change (%/year)	*n*	812	203	203	203	203		453
	Median (IQR)	1.03 (0.06)	0.99 (0.04)	1.01 (0.04)	1.04 (0.04)	1.07 (0.05)	** < 0.001**^**b**^	1.06 (0.05)
	Q1, Q3	1.00, 1.06	0.96, 1.01	0.99, 1.04	1.02, 1.06	1.05, 1.10		1.03, 1.08
	Mean ± SD	1.03 ± 0.06	0.99 ± 0.04	1.01 ± 0.03	1.04 ± 0.03	1.08 ± 0.06	** < 0.001**^**c**^	1.06 ± 0.05
Tolvaptan vs. placebo	LSM difference ± SE	− 0.027 ± 0.003	− 0.070 ± 0.004	− 0.043 ± 0.004	− 0.018 ± 0.004	0.023 ± 0.004	–	–
	*P* value^d^	** < 0.001**	** < 0.001**	** < 0.001**	** < 0.001**	** < 0.001**	–	–
	Mean difference (%)	− 2.535	− 6.641	− 4.043	− 1.662	2.205	–	–
Annual eGFR change over 3 years								
Annual change (ml/min per 1.73 m^2^/year)	*n*	812	203	203	203	203		453
	Median (IQR)	− 2.52 (3.64)	− 2.08 (3.40)	− 2.05 (3.09)	− 2.92 (3.44)	− 3.27 (4.53)	** < 0.001**^**b**^	− 3.49 (4.36)
	Q1, Q3	− 4.41, − 0.77	− 3.77, − 0.37	− 3.72, − 0.62	− 4.75, − 1.31	− 5.78, − 1.25		− 5.83, − 1.47
	Mean ± SD	− 2.60 ± 3.42	− 1.89 ± 2.67	− 2.12 ± 3.54	− 2.82 ± 3.17	− 3.55 ± 3.92	** < 0.001**^**c**^	− 3.64 ± 3.76
Tolvaptan vs. placebo	LSM difference ± SE	1.047 ± 0.208	1.748 ± 0.292	1.523 ± 0.312	0.825 ± 0.303	0.093 ± 0.322	–	–
	*P* value^d^	** < 0.001**	** < 0.001**	** < 0.001**	**0.007**	0.8	–	–
	Mean difference (%)	28.749	47.999	41.803	22.656	2.540	–	–

ANOVA, analysis of variance; eGFR, estimated glomerular filtration rate; eHTKV-α, estimated height-adjusted total kidney volume growth rate; IQR, interquartile range; LSM, least squares mean; SD, standard deviation; SE, standard error; TKV, total kidney volume; TQ, tolvaptan eHTKV-α quartile

^a^Analyses included all patients in TEMPO 3:4 with TKV data available to calculate eHTKV-α at baseline and year 1; tolvaptan-assigned patients were further divided into quartiles (TQ1–TQ4) based on percent change in eHTKV-α from baseline at 1 year

^b^*P* values were calculated using Pearson’s tests to determine whether there were significant correlations between change in eHTKV-α from baseline at 1 year and annual changes in TKV or eGFR over 3 years

^c^Statistical comparisons between tolvaptan eHTKV-α quartiles were based on ANOVA trend tests

^d^Statistical comparisons with the placebo group were based on ANOVA trend tests

Statistically significant values are indicated in bold

The annual eGFR change over 3 years and change in eHTKV-α from baseline at 1 year were significantly negatively correlated (*P* < 0.001; Fig. 2b). The annual change in eGFR over 3 years was smaller in TQ1–TQ3 (statistically significant) and TQ4 (*P* = 0.8) than in the placebo group (Table 2 and Fig. 3b).

**Fig. 4:**
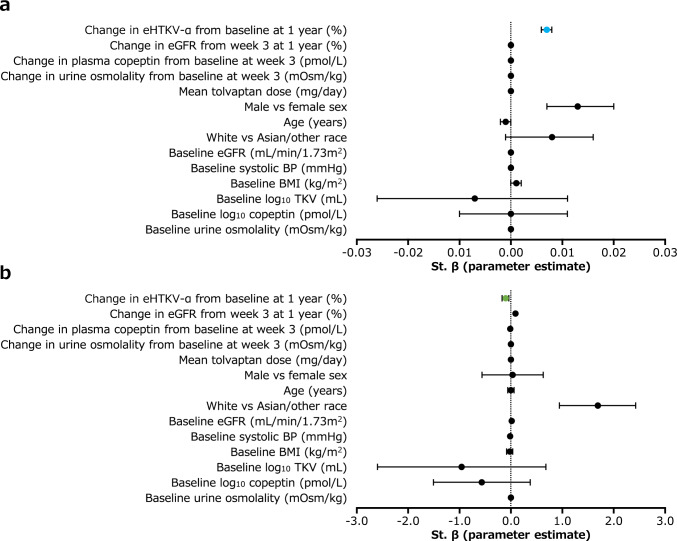
Predictive value of selected variables on annual changes in **a** TKV and **b** eGFR over 3 years. Data are based on multivariate regression analyses of tolvaptan-assigned patients in TEMPO 3:4 with TKV data available to calculate eHTKV-α at baseline and year 1 (*n* = 812); error bars represent 95% confidence intervals. BMI, body mass index; BP, blood pressure; eGFR, estimated glomerular filtration rate; eHTKV-α, estimated height-adjusted total kidney volume growth rate; St. β, standardised regression coefficient; TKV, total kidney volume

Univariate regression analysis confirmed that change in eHTKV-α from baseline at 1 year was a significant predictor of annual change in TKV over 3 years (*P* < 0.001; Table 3). This relationship remained significant in the multivariate regression analysis (*P* < 0.001; Table 3 and Fig. 4a) and after using backward stepwise selection as a variable reduction method (*P* < 0.001; Table 3). Multivariate regression analysis found that the change in plasma copeptin from baseline at week 3 was significantly associated with annual TKV change (*P* = 0.02), but baseline plasma copeptin, baseline urine osmolality and change in urine osmolality from baseline at week 3 were not associated with annual TKV change (Table 3). In addition to 1-year change in eHTKV-α, other significant predictors of annual change in TKV identified from the multivariate regression model using backward selection were sex, age and baseline body mass index.

**Table 3:** Predictive value of eHTKV-α and other variables for annual change in TKV over 3 years

Variable	Univariate regression model			Multivariate regression model			Multivariate regression model (variable reduction method)^a^		
	St. β (parameter estimate)	95% CI	*P* value	St. β (parameter estimate)	95% CI	*P* value	St. β (parameter estimate)	95% CI	*P* value
Change in eHTKV-α from baseline at 1 year (%)	0.009	0.008, 0.009	** < 0.001**	0.007	0.007, 0.008	** < 0.001**	0.009	0.008, 0.009	** < 0.001**
Change in eGFR from week 3 at 1 year (%)	− 0.000	− 0.001, − 0.000	**0.008**	0.000	− 0.000, 0.000	0.7	–	–	–
Change in plasma copeptin from baseline at week 3 (pmol/l)	− 0.000	− 0.000, 0.000	0.4	0.000	0.000, 0.000	**0.02**	–	–	–
Change in urine osmolality from baseline at week 3 (mOsm/kg)	− 0.000	− 0.000, 0.000	0.4	− 0.000	− 0.000, 0.000	0.2	–	–	–
Mean tolvaptan dose (mg/day)	0.000	− 0.000, 0.000	0.4	0.000	− 0.000, 0.000	0.9	–	–	–
Male vs. female sex	0.031	0.024, 0.039	** < 0.001**	0.013	0.007, 0.020	** < 0.001**	0.010	0.004, 0.016	**0.001**
Age (years)	− 0.002	− 0.002, − 0.001	** < 0.001**	− 0.001	− 0.002, − 0.001	** < 0.001**	− 0.002	− 0.002, − 0.001	** < 0.001**
White vs. Asian/other race	0.007	− 0.004, 0.017	0.2	0.008	− 0.001, 0.016	0.07	–	–	–
Baseline eGFR (ml/min per 1.73 m^2^)	− 0.000	− 0.000, 0.000	0.4	− 0.000	− 0.000, 0.000	0.6	–	–	–
Baseline systolic BP (mmHg)	0.000	− 0.000, 0.001	0.07	− 0.000	− 0.000, 0.000	0.9	–	–	–
Baseline BMI (kg/m^2^)	0.002	0.002, 0.003	** < 0.001**	0.001	0.000, 0.002	**0.002**	0.001	0.001, 0.002	** < 0.001**
Baseline log_10_ TKV (ml)^b^	0.044	0.025, 0.064	** < 0.001**	− 0.007	− 0.026, 0.011	0.4	–	–	–
Baseline log_10_ copeptin (pmol/l)^b^	0.015	0.002, 0.029	**0.02**	0.000	− 0.010, 0.011	0.9	–	–	–
Baseline urine osmolality (mOsm/kg)	− 0.000	− 0.000, 0.000	0.7	− 0.000	− 0.000, 0.000	0.6	–	–	–
R^2^	–	–	–	0.526^c^	–	–	0.535^c^	–	–

Regression analyses included tolvaptan-assigned patients in TEMPO 3:4 with TKV data available to calculate eHTKV-α at baseline and 1 (*n* = 812); statistically significant values are indicated in bold. Annual change in TKV (left and right kidneys) was calculated as the slope of a regression model over time on TKV values obtained at baseline, and years 1, 2 and 3

BMI, body mass index; BP, blood pressure; CI, confidence interval; eGFR, estimated glomerular filtration rate; eHTKV-α, estimated height-adjusted total kidney volume growth rate; R^2^, coefficient of determination; St. β, standardised regression coefficient; TKV, total kidney volume

^a^Backward stepwise selection was applied as the variable reduction method

^b^Log transformation was applied because baseline TKV and copeptin data are not normally distributed

^c^R^2^ represents the extent to which the variance of the dependent variables accounts for the variance of independent variables in a regression model

Similarly, univariate regression analysis confirmed that the change in eHTKV-α from baseline at 1 year was a significant predictor of annual change in eGFR over 3 years (*P* = 0.002; Table 4). This relationship remained significant in the multivariate analysis (*P* = 0.002; Table 4 and Fig. 4b) and after using backward stepwise selection as a variable reduction method (*P* < 0.001; Table 4). Multivariate regression analysis found that baseline urine osmolality was significantly associated with annual eGFR change (*P* = 0.03), but plasma copeptin (at baseline and change from baseline at week 3) and change in urine osmolality from baseline at week 3 were not associated with annual eGFR change (Table 4). In addition to the 1-year change in eHTKV-α, other significant predictors of annual change in eGFR identified from the multivariate regression model using backward selection were race, baseline eGFR and percent change in eGFR from week 3 at 1 year.

**Table 4:** Predictive value of eHTKV-α and other variables for annual change in eGFR over 3 years

Variable	Univariate regression model			Multivariate regression model			Multivariate regression model (variable reduction method)^a^		
	St. β (parameter estimate)	95% CI	*P* value	St. β (parameter estimate)	95% CI	*P* value	St. β (parameter estimate)	95% CI	*P* value
Change in eHTKV-α from baseline at 1 year (%)	− 0.119	− 0.195, − 0.043	**0.002**	− 0.101	− 0.164, − 0.038	**0.002**	− 0.103	− 0.155, − 0.050	** < 0.001**
Change in eGFR from week 3 at 1 year (%)	0.124	0.104, 0.144	** < 0.001**	0.093	0.071, 0.114	** < 0.001**	0.119	0.099, 0.140	** < 0.001**
Change in plasma copeptin from baseline at week 3 (pmol/l)	− 0.015	− 0.044, 0.014	0.3	− 0.014	− 0.030, 0.003	0.1	–	–	–
Change in urine osmolality from baseline at week 3 (mOsm/kg)	− 0.001	− 0.003, 0.001	0.3	0.002	− 0.001, 0.004	0.2	–	–	–
Mean tolvaptan dose (mg/day)	− 0.003	− 0.022, 0.016	0.8	− 0.003	− 0.015, 0.009	0.6	–	–	–
Male vs. female sex	0.143	− 0.665, 0.951	0.7	0.036	− 0.560, 0.633	0.9	–	–	–
Age (years)	0.003	− 0.055, 0.061	0.9	− 0.000	− 0.044, 0.043	0.9	–	–	–
White vs. Asian/other race	1.219	0.124, 2.314	**0.03**	1.687	0.947, 2.427	** < 0.001**	1.193	0.541, 1.846	** < 0.001**
Baseline eGFR (ml/min per 1.73 m^2^)	0.018	− 0.001, 0.037	0.07	0.015	− 0.002, 0.032	0.09	0.018	0.007, 0.030	**0.002**
Baseline systolic BP (mmHg)	0.000	− 0.030, 0.030	0.9	− 0.017	− 0.037, 0.002	0.08	–	–	–
Baseline BMI (kg/m^2^)	− 0.013	− 0.095, 0.068	0.7	− 0.019	− 0.074, 0.035	0.5	–	–	–
Baseline log_10_ TKV (ml)^b^	− 2.128	− 4.173, − 0.082	**0.04**	− 0.953	− 2.589, 0.683	0.3	–	–	–
Baseline log_10_ copeptin (pmol/l)^b^	− 1.419	− 2.823, − 0.014	**0.05**	− 0.563	− 1.503, 0.377	0.2	–	–	–
Baseline urine osmolality (mOsm/kg)	0.003	0.000, 0.005	**0.03**	0.003	0.000, 0.006	**0.03**	–	–	–
R^2^	–	–	–	0.234^c^	–	–	0.198^c^	–	–

Regression analyses included tolvaptan-assigned patients in TEMPO 3:4 with TKV data available to calculate eHTKV-α at baseline and year 1 (*n* = 812); statistically significant values are indicated in bold. Annual change in eGFR was calculated as the slope of a regression model over time across all on-treatment eGFR values from week 3 through year 3

BMI, body mass index; BP, blood pressure; CI, confidence interval; eGFR, estimated glomerular filtration rate; eHTKV-α, estimated height-adjusted total kidney volume growth rate; R^2^, coefficient of determination; St. β, standardised regression coefficient; TKV, total kidney volume

^a^Backward stepwise selection was applied as the variable reduction method

^b^Log transformation was applied because baseline TKV and copeptin data are not normally distributed

^c^R^2^ represents the extent to which the variance of the dependent variables accounts for the variance of independent variables in a regression model

## Discussion

This post hoc analysis of the TEMPO 3:4 trial revealed that the 1-year change in eHTKV-α was a strong predictor of tolvaptan efficacy in patients with ADPKD. In tolvaptan-treated patients, percent change in eHTKV-α from baseline at 1 year was significantly associated with annual changes in TKV and eGFR from baseline over 3 years, suggesting that early changes in eHTKV-α may be a clinically useful predictor of treatment response and longer-term functional outcomes. As such, detection of early percent decreases in eHTKV-α would allow for prompt identification of patients who are likely to have a good response to tolvaptan treatment by using eHTKV-α quartile.

Data from the landmark Consortium of Radiologic Imaging Studies of Polycystic Kidney Disease (CRISP) study previously revealed that TKV increased exponentially and TKV growth was associated with renal function decline [21]. In the CRISP follow-up study, it was demonstrated that HtTKV predicted the risk of developing renal insufficiency, highlighting HtTKV as a potential prognostic biomarker [22]. These data informed the Mayo Imaging Classification system, which uses HtTKV and age to predict renal prognosis, and thus identifies rapidly progressing patients for treatment and inclusion in clinical trials [16]. The Mayo-imaging classification is not designed to estimate the effect of treatment on ADPKD progression; therefore, subsequent studies have suggested eHTKV-α as a potential predictor of treatment response [20]. Consistent with these findings, this post hoc analysis of the TEMPO 3:4 trial demonstrated that greater percent reductions in eHTKV-α after 1 year of tolvaptan therapy were significantly associated with slower rates of TKV growth and eGFR decline over 3 years of treatment.

In TEMPO 3:4, the effect of tolvaptan on TKV growth was most pronounced in the first year of treatment and likely due to an acute reduction in the secretion of cyst fluid [6]. Stable eHTKV-α over 3 years is consistent with the view that cyst growth suppression with tolvaptan is stable over 3 years, which may not be produced without the inhibition of cyst cell proliferation.

Although the annual increase in TKV over 3 years in TQ4 was statistically higher in tolvaptan-treated than in placebo-treated patients, the annual eGFR change over 3 years was not significantly different. The effect of TKV growth suppression on renal function decline is expected to be influenced by heterogeneous eGFR trajectories [12], sex [20, 23], body mass index [24], and race [25] in patients with ADPKD; these factors were significantly different among eHTKV-α quartiles. In addition, the weak association between the annual change in TKV and eGFR in TQ4 may also relate to direct tolvaptan effect to slow renal function decline which is independent of tolvaptan effect of renal cyst growth suppression [26].

While several predictors of tolvaptan response in TEMPO 3:4 have previously been identified, including higher urine osmolality and plasma copeptin levels at baseline, greater suppression of urine osmolality and a greater increase in plasma copeptin from baseline at week 3 [13, 14], our data suggest that change in eHTKV-α at 1 year is a comparatively stronger predictor of treatment outcomes. Moreover, measurements of urine osmolality and plasma copeptin are not widely integrated into the routine management of patients with ADPKD. On the other hand, TKV measurement is already regularly performed to estimate ADPKD disease progression and identify suitable patients for tolvaptan treatment [8–10].

The substantially larger decrease in eHTKV-α from baseline to tolvaptan treatment years was reported to be correlated significantly with baseline higher eHTKV-α [18]. Substantial vs percent change may have different physiological significance, but the relationship between substantial change in eHTKV-α and renal outcomes had not been examined because refining equation constants of eHTKV-α formula was a major focus of this report [18].

The selection of patients who are at high risk of progressing to ESRD, as well as the identification of patients who are likely to respond well to treatment, are recommended for better clinical practise [8–10]. eHTKV-α is a useful biomarker for identifying rapid progressors [19] and good responders, as shown in this study. Further prospective studies are needed to confirm the usefulness of 1-year change in eHTKV-α as a predictor of longer-term treatment efficacy in routine clinical practice.

The current analysis has the following limitations. In TEMPO 3:4, most patients had estimated creatine clearance of ≥ 60 ml/min [6], thus limiting the generalisability of these findings to patients with more advanced renal dysfunction. The multivariate analyses were adjusted for patient background; however, the effect of other potential confounding factors was not assessed.

In conclusion, this study found that 1-year percent change in eHTKV-α may represent a clinically useful biomarker of selecting patients most likely to benefit from long-term tolvaptan therapy and may encourage such patients with good response in eHTKV-α biomarker to continue tolvaptan therapy and may be utilized for early estimate of renal outcomes of clinical trials on new drugs expected to reduce TKV growth in ADPKD.

## Supplementary Information

Below is the link to the electronic supplementary material.

Supplementary Table S1: Additional baseline patient demographics and clinical parameters by tolvaptan eHTKV-α quartile. (DOCX 40 KB)

## Data Availability

Qualified researchers can submit inquiries related to Otsuka clinical research or request access to individual participant data associated with any Otsuka clinical trial at https://clinical-trials.otsuka.com/. For all approved individual participant data access requests, Otsuka will share anonymised individual participant data on a remotely accessible data sharing platform.
