# Supplementary material for: Change in kidney volume growth rate and renal outcomes of tolvaptan treatment in autosomal dominant polycystic kidney disease: post-hoc analysis of TEMPO 3:4 trial
Source: Clin Exp Nephrol. 2025 Jan 2;29(5):638–49. doi: 10.1007/s10157-024-02589-1 (PMC12049310; doi:10.1007/s10157-024-02589-1)
Supplement: Supplementary file 1 — Supplementary Table S1: Additional baseline patient demographics and clinical parameters by tolvaptan eHTKV-α quartile. (DOCX 40 KB) [file 10157_2024_2589_MOESM1_ESM.docx]

**Change in kidney volume growth rate and renal outcomes of tolvaptan treatment in autosomal dominant polycystic kidney disease: post-hoc analysis of TEMPO 3:4 trial**

**Supplementary File**

**Authors:** Eiji Higashihara, MD, PhD, Miyuki Matsukawa, MSc and Huan Jiang, PhD

**Correspondence:** Eiji Higashihara. E-mail: ehigashi@ks.kyorin-u.ac.jp

**Supplementary Table S1.** Additional baseline patient demographics and clinical parameters by tolvaptan eHTKV-α quartile

|  | **Tolvaptan^a^** | | | | | | **Placebo^a^** | |
| --- | --- | --- | --- | --- | --- | --- | --- | --- |
|  | **All**  **(*n* = 812)** | **Tolvaptan eHTKV-α quartile** | | | | ***P* value^b^** | **All**  **(*n* = 453)** | ***P* value^c^** |
|  |  | **TQ1**  **(*n* = 203)** | **TQ2**  **(*n* = 203)** | **TQ3**  **(*n* = 203)** | **TQ4**  **(*n* = 203)** |  |  |  |
| Baseline demographics |  |  |  |  |  |  |  |  |
| Race, *n* (%) |  |  |  |  |  |  |  |  |
| White | 682 (84.0) | 166 (81.8) | 172 (84.7) | 165 (81.3) | 179 (88.2) | 0.2 | 379 (83.7) | 0.9 |
| Asian | 106 (13.1) | 34 (16.7) | 27 (13.3) | 32 (15.8) | 13 (6.4) | **0.007** | 61 (13.5) | 0.8 |
| Other | 24 (3.0) | 3 (1.5) | 4 (2.0) | 6 (3.0) | 11 (5.4) | **0.02** | 13 (2.9) | 0.9 |
| Height (cm), mean ± SD | 173.5 ± 10.3 | 170.8 ± 9.8 | 173.6 ± 11.2 | 173.9 ± 10.6 | 175.5 ± 9.1 | **< 0.001** | 173.4 ± 9.6 | 0.9 |
| Weight (kg), mean ± SD | 79.2 ± 17.9 | 74.4 ± 18.2 | 77.7 ± 18.2 | 81.0 ± 18.2 | 83.7 ± 15.5 | **< 0.001** | 77.9 ± 17.7 | 0.2 |
| BMI (kg/m^2^), mean ± SD | 26.2 ± 5.0 | 25.3 ± 4.8 | 25.7 ± 5.0 | 26.7 ± 5.4 | 27.1 ± 4.4 | **< 0.001** | 25.8 ± 4.8 | 0.1 |
| Kidney function parameters |  |  |  |  |  |  |  |  |
| Urine creatinine (mg/dl), *n* | 810 | 202 | 203 | 202 | 203 |  | 453 |  |
| Mean ± SD | 89.6 ± 54.4 | 95.2 ± 55.2 | 90.6 ± 53.3 | 80.1 ± 48.5 | 92.3 ± 59.1 | **0.03** | 86.3 ± 52.4 | 0.3 |
| Estimated CrCl (ml/min), *n* | 809 | 203 | 202 | 201 | 203 |  | 451 |  |
| Mean ± SD | 103.3 ± 32.4 | 102.7 ± 30.5 | 102.4 ± 33.4 | 103.3 ± 35.0 | 104.7 ± 30.7 | 0.9 | 102.8 ± 34.7 | 0.8 |
| Urinary albumin-to-creatinine ratio (mg/mmol), *n* | 763 | 194 | 197 | 188 | 184 |  | 424 |  |
| Mean ± SD | 7.4 ± 15.5 | 7.4 ± 14.1 | 8.5 ± 16.5 | 7.4 ± 16.7 | 6.2 ± 14.6 | 0.6 | 8.7 ±22.1 | 0.3 |
| BP parameters |  |  |  |  |  |  |  |  |
| Systolic BP (mmHg), *n* | 812 | 203 | 203 | 203 | 203 |  | 453 |  |
| Mean ± SD | 128.4 ± 13.6 | 127.4 ± 14.1 | 129.0 ± 14.5 | 128.8 ± 13.1 | 128.3 ± 12.6 | 0.6 | 128.5 ± 13.4 | 0.9 |
| Diastolic BP (mmHg), *n* | 812 | 203 | 203 | 203 | 203 |  | 453 |  |
| Mean ± SD | 82.3 ± 9.8 | 81.5 ± 10.4 | 82.7 ± 10.0 | 82.7 ± 9.8 | 82.5 ± 9.1 | 0.6 | 82.5 ± 9.3 | 0.8 |

ANOVA, analysis of variance; BMI, body mass index; BP, blood pressure; CrCl, creatinine clearance; eHTKV-α, estimated height-adjusted total kidney volume growth rate; SD, standard deviation; TQ, tolvaptan eHTKV-α quartile.

^a^Analyses included all patients in TEMPO 3:4 with TKV data available to calculate eHTKV-α at baseline and month 12; tolvaptan-assigned patients were further divided into quartiles (TQ1–TQ4) based on percent change in eHTKV-α from baseline at 1 year.

^b^Statistical comparisons between tolvaptan eHTKV-α quartiles were based on Cochran Armitage tests for categorical variables and ANOVA trend tests for continuous variables.

^c^Statistical comparisons between the overall tolvaptan and placebo treatment groups were based on Chi-squared tests for categorical variables and two-sample t-tests for continuous variables.

Statistically significant values are indicated in bold.


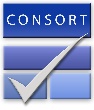
CONSORT 2010 checklist of information to include when reporting a randomised trial*

| Section/Topic | Item No | Checklist Item | Reported on Page No |
| --- | --- | --- | --- |
| Title and abstract | | | |
|  | 1a | Identification as a randomised trial in the title | 1 and Graphical abstract |
|  | 1b | Structured summary of trial design, methods, results, and conclusions (for specific guidance see CONSORT for abstracts) | 2 (+ ‘Abstract’ in primary publication) |
| Introduction | | | |
| Background and objectives | 2a | Scientific background and explanation of rationale | 3 (Key learning points) and 5-6 |
|  | 2b | Specific objectives or hypotheses | 6 (+ Key learning points) |
| Methods | | | |
| Trial design | 3a | Description of trial design (such as parallel, factorial) including allocation ratio | 6-7 (+ ‘Methods’ in primary publication) |
|  | 3b | Important changes to methods after trial commencement (such as eligibility criteria), with reasons | NA |
| Participants | 4a | Eligibility criteria for participants | 2, 6-7 (+ ‘Methods’ in primary publication) |
|  | 4b | Settings and locations where the data were collected | See ‘Methods’ in primary publication |
| Interventions | 5 | The interventions for each group with sufficient details to allow replication, including how and when they were actually administered | 6-7, 9 (+ ‘Methods’ in primary publication |
| Outcomes | 6a | Completely defined pre-specified primary and secondary outcome measures, including how and when they were assessed | 8 and 9 (+ ‘Methods’ in primary publication) |
|  | 6b | Any changes to trial outcomes after the trial commenced, with reasons | NA |
| Sample size | 7a | How sample size was determined | See ‘Statistical analysis’ in primary publication |
|  | 7b | When applicable, explanation of any interim analyses and stopping guidelines | NA |
| Randomisation: |  |  |  |
| Sequence generation | 8a | Method used to generate the random allocation sequence | See ‘Methods’ in primary publication |
|  | 8b | Type of randomisation; details of any restriction (such as blocking and block size) | NA |
| Allocation concealment mechanism | 9 | Mechanism used to implement the random allocation sequence (such as sequentially numbered containers), describing any steps taken to conceal the sequence until interventions were assigned | NA |
| Implementation | 10 | Who generated the random allocation sequence, who enrolled participants, and who assigned participants to interventions | See ‘Methods’ in primary publication |
| Blinding | 11a | If done, who was blinded after assignment to interventions (for example, participants, care providers, those assessing outcomes) and how | NA |
|  | 11b | If relevant, description of the similarity of interventions | NA |
| Statistical methods | 12a | Statistical methods used to compare groups for primary and secondary outcomes | 8-9 (+ ‘Statistical analysis’ in primary publication) |
|  | 12b | Methods for additional analyses, such as subgroup analyses and adjusted analyses | 7-9 |
| Results | | | |
| Participant flow (a diagram is strongly recommended) | 13a | For each group, the numbers of participants who were randomly assigned, received intended treatment, and were analysed for the primary outcome | 9 (+ ‘Figure 1’ in primary publication) |
|  | 13b | For each group, losses and exclusions after randomisation, together with reasons | See ‘Figure 1’ in primary publication |
| Recruitment | 14a | Dates defining the periods of recruitment and follow-up | 7 (+ ‘Methods’ in primary publication) |
|  | 14b | Why the trial ended or was stopped | NA |
| Baseline data | 15 | A table showing baseline demographic and clinical characteristics for each group | 9–10, Table 1, Supplementary Table S1 (+ ‘Table 1’ in primary publication) |
| Numbers analysed | 16 | For each group, number of participants (denominator) included in each analysis and whether the analysis was by original assigned groups | 9 (Table 1) |
| Outcomes and estimation | 17a | For each primary and secondary outcome, results for each group, and the estimated effect size and its precision (such as 95% confidence interval) | 9 and Table 1(+ ‘Results’ of primary publication) |
|  | 17b | For binary outcomes, presentation of both absolute and relative effect sizes is recommended | NA |
| Ancillary analyses | 18 | Results of any other analyses performed, including subgroup analyses and adjusted analyses, distinguishing pre-specified from exploratory | 9–11, Tables 2–4, Figures 1–4 |
| Harms | 19 | All important harms or unintended effects in each group (for specific guidance see CONSORT for harms) | NA |
| Discussion | | | |
| Limitations | 20 | Trial limitations, addressing sources of potential bias, imprecision, and, if relevant, multiplicity of analyses | 13 (+ ‘Discussion’ in primary publication) |
| Generalisability | 21 | Generalisability (external validity, applicability) of the trial findings | 3 (Potential impact of Key learning points)  And 11-13 |
| Interpretation | 22 | Interpretation consistent with results, balancing benefits and harms, and considering other relevant evidence | 11-13 |
| Other information | | |  |
| Registration | 23 | Registration number and name of trial registry | 6-7 |
| Protocol | 24 | Where the full trial protocol can be accessed, if available | See ‘Methods’ of primary publication |
| Funding | 25 | Sources of funding and other support (such as supply of drugs), role of funders | 14 |

Citation: Schulz KF, Altman DG, Moher D, for the CONSORT Group. CONSORT 2010 Statement: updated guidelines for reporting parallel group randomised trials. BMC Medicine. 2010;8:18.
© 2010 Schulz et al. This is an Open Access article distributed under the terms of the Creative Commons Attribution License (<http://creativecommons.org/licenses/by/2.0>), which permits unrestricted use, distribution, and reproduction in any medium, provided the original work is properly cited.

*We strongly recommend reading this statement in conjunction with the CONSORT 2010 Explanation and Elaboration for important clarifications on all the items. If relevant, we also recommend reading CONSORT extensions for cluster randomised trials, non-inferiority and equivalence trials, non-pharmacological treatments, herbal interventions, and pragmatic trials. Additional extensions are forthcoming: for those and for up-to-date references relevant to this checklist, see [www.consort-statement.org](http://www.consort-statement.org).
